# Circulating microRNAs in Medicine

**DOI:** 10.3390/ijms23073996

**Published:** 2022-04-03

**Authors:** Tetiana Pozniak, Dzmitry Shcharbin, Maria Bryszewska

**Affiliations:** 1 Institute of Biophysics and Cell Engineering of the National Academy of Sciences of Belarus, 220072 Minsk, Belarus; 2 Palladin Institute of Biochemistry, National Academy of Sciences of Ukraine, 02000 Kyiv, Ukraine; 3 Department of General Biophysics, Faculty of Biology and Environmental Protection, University of Lodz, 90-236 Lodz, Poland; maria.bryszewska@biol.uni.lodz.pl

**Keywords:** circulating microRNAs (c-microRNAs, c-miRNAs), small interfering RNAs (miRNAs), oncology, cardiovascular diseases, neurodegenerative diseases

## Abstract

Circulating microRNAs (c-microRNAs, c-miRNAs), which are present in almost all biological fluids, are promising sensitive biomarkers for various diseases (oncological and cardiovascular diseases, neurodegenerative pathologies, etc.), and their signatures accurately reflect the state of the body. Studies of the expression of microRNA markers show that they can enable a wide range of diseases to be diagnosed before clinical symptoms are manifested, and they can help to assess a patient’s response to therapy in order to correct and personalize treatments. This review discusses the latest trends in the uses of miRNAs for diagnosing and treating various diseases, viral and non-viral. It is concluded that exogenous microRNAs can be used as high-precision therapeutic agents for these purposes.

## 1. Introduction

More than 20 years ago, a mechanism of negative regulation of gene expression at the level of translation was discovered: mRNA is blocked by small non-coding RNAs, repressing translation or promoting mRNA degradation. This process has been called RNA interference and it leads to “gene silencing”. Several subclasses of small non-coding RNAs (snRNAs) are involved in this powerful post-transcriptional gene silencing process. RNA interference was first described by Andrew Fire and Craig Mello in *Nature* in 1998. They received the Nobel Prize in Physiology or Medicine (2006) for this discovery [1]. They showed that short double-stranded RNAs can silence homologous genes.

There are many groups of snRNAs and the list is growing rapidly. This review details the components of RNA interference, such as microRNAs (miRNAs) and small interfering RNAs (siRNAs). These two species have similar structures and are short (21–23 and 20–24 nucleotides, respectively) single- and double-stranded RNAs that inhibit gene expression. The main differences between them lie in their mechanisms of formation and their degrees of homology with respect to targeting of mRNAs.

In 1993, it was determined that the *lin-4* gene in *Caenorhabditis elegans* produced a snRNA [2,3]. In 1998, *lin-4* was shown to encode a 61-nucleotide precursor that matured into a 22-nucleotide RNA, later called miRNA. This short RNA contains sequences that are partially complementary to the 3′-untranslated region (3′-UTR) of mRNA transcribed from the *lin-14* nematode gene and represses translation of this mRNA, inhibiting LIN-14 protein synthesis. In 2000, a second miRNA was discovered, a product of *let-7* [2], which suppressed the expression of several genes simultaneously and was later identified in a number of organisms, including humans. Although *lin-4* and *let-7* were identified by standard positional cloning of genetic loci, most miRNA genes are detected by cloning cDNA sequences complementary to the desired RNA fragments. This method involves the isolation of a miRNA that blocks the translation of a specific messenger, followed by cDNA synthesis using reverse transcriptase. One difficulty in finding miRNA genes for further cDNA cloning is that not only fragments of the target but also fragments of other noncoding RNAs (such as rRNA, tRNA, and snRNA), together with mRNAs, are cloned from RNA samples of a selected size. However, this difficulty is easily solved by comparing the candidate miRNA sequence with known miRNA sequences in annotated databases [4]. To date, more than 2000 miRNAs have been registered in this database. 

There are certain rules for miRNA nomenclature [5,6]: (1) all miRNAs are abbreviated as miR; (2) newly discovered miRNAs are assigned sequential numerical identifiers (for example, miR5, miR6, miR7); (3) to designate the species of origin of a miRNA, a prefix of 3–4 letters can be added to the name (*hsa* for *Homo sapiens*, *dme* for *Drosophila melanogaster*, etc.); (4) orthologous miRNAs from different species are given parallel names (*hsa-miR-28, ptr-mir-28, crf-miR-28*); (5) paralogous miRNAs with one or two different bases are distinguished by suffixes (e.g., miR-10a and miR-10b); (6) if identical miRNAs originate from separate loci in the same organism, they are assigned numerical suffixes (for example, *miR*-281-1 and *miR*-281-2 in *Drosophila melanogaster*).

## 2. miRNA Biogenesis

**Figure 1:**
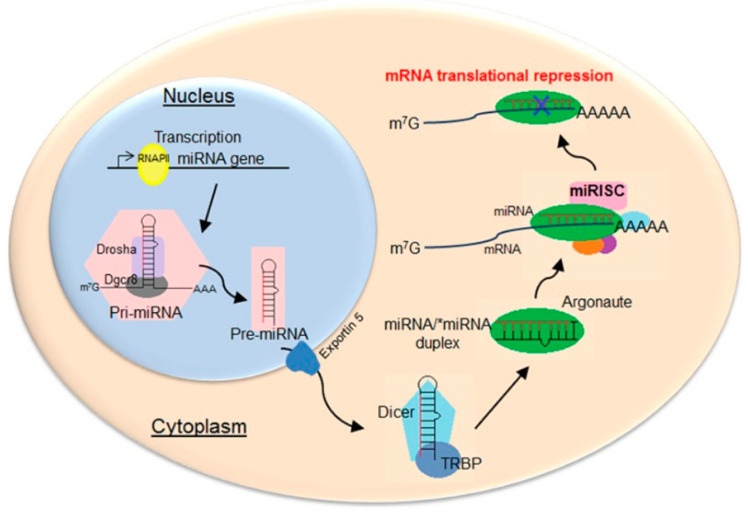
Scheme of biogenesis and functions of miRNAs in animals. Reprinted/adapted with permission from Ref. [14], 2015, Creative Commons Attribution-NonCommercial 4.0 International License.

The miRNA precursors, pre-miRNAs, are first transcribed as capped polyadenylated strands that form double-stranded stem–loop structures (Figure 1). In the nucleus, these transcripts are processed by the Drosha enzyme of the RNase III family to form pre-miRNAs of 70–100 nucleotides with a hairpin structure (two base pairs connected by a loop), a 5′-phosphate group, and a 3′-double nucleotide [7]. The pre-miRNAs are exported to the cytoplasm via exportin-5 [8]; microRNAs of 21–23 nucleotide sequences are formed from them by enzymes, including the Dicer ribonuclease complex [9]. This is part of the RISC (RNA-Induced Silencing Complex) that unwinds miRNA chains, cleaving and releasing the “passenger” chain and the guide chain. Argonaute 2 endonuclease [10,11], which is also part of the complex, interacts with complementary regions of mRNA, leading to degradation of the latter and/or blocking of translation [12,13].

siRNA is formed from its double-stranded RNA (dsRNA) precursor in the same way as miRNA. However, an important distinguishing feature is that siRNAs are only partially complementary to the 3′UTR region of their target mRNAs, so they can inactivate several different mRNAs simultaneously [15]. They regulate their targets through four Argonaute proteins, and although they sometimes induce mRNA cleavage and degradation as miRNAs do, they primarily mediate gene silencing through translational repression and mRNA degradation through deadenylation [16]. As a rule, miRNAs pair accurately with their targets and promote endonucleotic cleavage of a single specific mRNA [17].

## 3. Functions of miRNA

**Figure 2:**
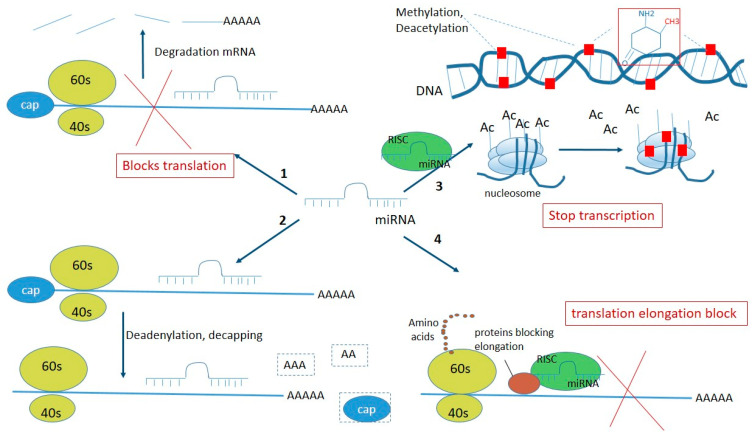
Scheme of inhibition of gene expression by miRNA in several ways (description in the article). Ac—acetyl, CH_3_CO.

The modern scientific approach to the search for therapeutic agents is to develop drugs that selectively influence processes at the gene level. The main targets of this approach are non-coding double-stranded miRNAs because they cause gene silencing by direct interaction with mRNA, suppressing the production of proteins in various diseases (for example, anti-apoptotic proteins in malignant neoplasms [18] and anti-miRNA that inhibits the activity of miRNA-122 in the liver in hepatitis C patients [19]). snRNAs regulate the expression of more than 30% of the protein-encoding genes in humans [20]. They inhibit gene expression in several ways [21]: (1) by interacting with mRNA, directly binding to the target, leading to blocking of translation (protein synthesis) and to mRNA degradation (if there is perfect complementary pairing, which is more characteristic of miRNAs); (2) mRNA deadenylation; (3) at the level of transcription, when snRNAs within the polyprotein complex cause epigenetic modifications of the genome—DNA methylation, deacetylation, and histone methylation; (4) by interaction with repressor proteins, blocking translation at the level of elongation (Figure 2) [22]. Additionally, when the cell cycle stops, miRNAs can activate as well as repress translation. This phenomenon was described in 2007 in *Science* [23]. Through combinations of these mechanisms, snRNAs affect protein synthesis in all cells and are significant in cellular processes, such as differentiation, proliferation, apoptosis, and metabolism. The biological processes can be regulated at several levels and lead to a decrease or increase in the number of miRNAs in a cell. Deregulation of miRNA expression can be genetic and result from chromosomal loss (deletion), amplification, translocation, or even point mutations of genes. miRNA expression can be affected by cytosine methylation in DNA, with hypermethylation or hypomethylation of CpG regions, post-translational modifications of histones in many types of tumors, and a decrease in transcription factors, such as p53 and c-Myc. Anomalies in proteins involved in different stages of the maturation process can also disrupt snRNA expression; for example, mutations affecting binding to the Drosha protein, miRNA export from the nucleus to the cytoplasm via exportin-5, or interaction with the Dicer enzyme [24].

In the mammalian genome, a single miRNA can regulate many genes, binding to more than 60% of the mRNAs involved in a specific signaling cascade or cellular mechanism. This makes them effective biological regulators capable of targeted suppression of “disease-causing” genes.

In 2001, it was demonstrated that the introduction of chemically synthesized miRNAs into mammalian cells effectively inhibits gene expression [25]. This discovery inspired many further studies of the “silencing” of gene expression through RNA interference. It has been established that miRNAs are involved in the regulation of many cellular functions, and aberrant miRNA expression leads to various disorders, cancer in particular, correlating with its various types [26,27]. miRNAs are involved in almost every stage of carcinogenesis: cell growth, differentiation, proliferation, angiogenesis, apoptosis, invasion, and metastasis [28]. Changes in expression can result from mutation, methylation, deletion, and amplification of miRNA-coding regions. In this case, miRNAs are either oncogenic and are activated, or function by acting on the mRNAs of tumor suppressors or oncogenes. For example, miRNA-suppressors are suppressed in cancer. The study of miRNA expression profiles enables normal tissues to be distinguished from cancerous ones, tissues of origin to be identified, and highly accurate information on the subtype of a particular cancer to be provided [29,30]. No less important is the fact that miRNA profiles can be used to predetermine the response to therapy and the further development of the disease. For example, miRNA expression can be used to predict a patient’s individual response to a drug [27,31].

Studies of miRNAs that affect the enzymes involved in processing have shown that they also control the differentiation, post-meiotic function, and growth of male germ cells, and the development and maturation of oocytes, through highly regulated gene expression. The expression of miRNA genes at different stages of testicular and ovarian cell development indicates a potential role in the physiology of the genital organs [32].

Changes in miRNA expression lead not only to cancer [29] but also to many other pathological processes, such as cardiovascular diseases [33,34], autoimmune diseases [35,36], disorders of the central nervous system [37], viral respiratory diseases [38], etc. It is not surprising that scientists around the world have focused attention on miRNAs as promising tools for highly selective post-transcriptional inhibition of gene expression.

## 4. Circulating miRNAs (c-miRNAs) and Their Differences

All organisms transmit genetic information from parent to offspring through vertical gene transfer. Bacteria also have horizontal (lateral) gene transfer for exchanging genetic information, which allows them to diversify their populations and facilitate adaptation to new conditions. Horizontal information transfer in eukaryotes was discovered relatively recently through the intercellular transport of miRNAs [39]. In 2008, circulating miRNA (c-microRNA) was first detected in maternal plasma [40]. Later, c-microRNAs were also found in other biological fluids, such as blood (serum, plasma), urine, cerebrospinal fluid, saliva, milk, lacrimal and seminal fluids, and bronchial lavage [41,42,43]. The appearance of c-microRNAs in the blood can result from secretion by cells or from cell death during apoptosis, necrosis, tumors, or trauma.

Interestingly, despite the presence of various nucleases, circulating endogenous miRNAs remain stable while pure exogenous miRNAs added to plasma degrade rapidly [41]. Subsequent studies established that endogenous miRNAs are highly stable and resistant not only to endogenous ribonucleases but also to extreme temperatures, pH levels, and freeze–thaw cycles [44]. To ascertain what protects them from enzymatic degradation, let us consider the mechanisms of miRNA entry into extracellular fluids and the forms in which they are found there. Initially, c-miRNAs can be secreted into the extracellular space in microvesicles (late endosomal compartments [45]), ectosomes [46], exosomes [47], or microvesicles (liposomes), or they can be associated with high-density lipoproteins (HDL) [48], or released as parts of apoptotic bodies. However, most of them, according to Reiner et al., form complexes with the proteins AGO2 or NPM1 (nucleophosmin 1) [49]. Regardless of the form in which they enter the extracellular space, miRNAs then pass into other biological fluids, for example, the general bloodstream.

Microvesicles (microparticles or ectosomes) are plasma membrane-derived particles released into the extracellular space by budding out and detachment from the plasma membrane. They range in size from 100 nm to 1 µm and are formed by outward protrusion of the plasma membrane, followed by separation [50]. Ectosomes are secreted by various cells, including tumor cells, polymorphonuclear leukocytes, aging erythrocytes, and activated platelets. One of their characteristic features is the appearance of phosphatidylserine (PS) on their membrane surfaces. Unlike exosomes, ectosomes bind well to annexin V and can also bind to prothrombin and blood coagulation factor X to form the prothrombinase complex [41].

Exosomes are small, membrane-bound vesicles of endosomal origin, 30–100 nm in diameter, that form intracellularly via endocytic invagination and are released into a structure known as the multivesicular body (MVB). The MVB then fuses with the plasma membrane, releasing the exosome contents into the extracellular space [51]. Exosomes are secreted by almost all normal cells (T-cells, B-lymphocytes, dendritic cells, reticulocytes, neurons, intestinal epithelial cells, platelets, etc.) and by pathological cells. It was previously believed that exosomes function only as protein scavengers, but in 2007, Valadi et al. found that they can carry nucleic acids (NAs), in particular, RNA [52]. It is now known that they can contain various components of their donor cells, including proteins (proteolytic proteins, chaperones (Hsp70 and Hsp90), apoptotic proteins (Alix), translation factors, metabolic enzymes), lipids, mRNA, microRNA, small interfering RNA (siRNA), and DNA. They can also carry viruses and prions from an infected cell [53]. Thus, exosomes function as horizontal carriers (between cells of the same organism, donor–recipient) of information, mRNA, viruses, and other materials, such as proteins and microRNAs. Some proteins are exosome-specific and do not depend on the donor cell; however, there are also specific ones, so it is possible to identify a subpopulation of specific exosomes and infer the cell type of origin [54]. However, how the various components making up exosomes are chosen remains an open question. In 2012, 4563 proteins had been found in exosomes [55], and the list of exosomal miRNAs numbered about 800. Cells can interact with each other by transferring exosomes loaded with miRNAs [56,57]. The authors of [57] showed that monocyte exosomes deliver miR-150 to endothelial cells and enhance their migration by reducing c-myb 9 expression. The miRNA content of exosomes is critical in this type of intercellular communication and determines the fate of the recipient cell. Thus, exosomes derived from mesenchymal stromal cells of the bone marrows of myeloma patients promote tumor growth, and this effect depends on their miR-15a content.

Exosomes produced by stressed cells also provide information that induces resistance in surrounding cells through a paracrine effect. For example, miRNAs regulate the activity of pancreatic β-cells by transfer via exosomes [58]. Exosomes are also involved in transmitting the immune response; miRNAs transferred by exosomes from T-cells to antigen-presenting cells (APCs) can regulate gene expression in the recipient cells [59]. Exactly this process accounts for the suppression of antitumor immunity, including inhibition of T-lymphocyte and natural killer activities, and the suppression of APC differentiation. Tumor exosomes also enhance the activity of immunosuppressive cells and increase their number. There are indications that tumor cells dispose of chemotherapy drugs (in particular, doxorubicin) by secreting them in exosomes. This process underlies the acquisition of resistance to anticancer therapy by malignant cells [60]. Considering these properties, exosomes can be considered promising tools for delivering drugs and engineering exogenous miRNAs for high-precision treatment of various diseases. Details about exosomes, their functions, mechanisms of action, and clinical applications have been reviewed [53,61].

In the extracellular fluid, some c-miRNAs are complexed with HDL [24,48]. Cellular export of miRNAs to HDL is regulated by neutral sphingomyelinase. The authors have demonstrated that reduced HDLs injected into mice form complexes with various miRNAs in normal and atherogenic models. The HDL-miRNA profile differs significantly between a healthy person and one with hereditary hypercholesterolemia. It is noteworthy that HDL-miRNAs in atherosclerosis induce differential gene expression with a significant loss of conserved mRNA targets in cultured hepatocytes. Taken together, these observations indicate that HDL is involved in intercellular communication, including the transport and delivery of miRNAs.

Other vesicles that transport miRNAs in the intercellular fluid are apoptotic bodies. These are vesicles with a diameter of 50 nm to 4 μm, formed from cells undergoing apoptosis [62].

The foregoing shows that c-miRNAs function as secreted signaling molecules and affect the phenotypes of recipient cells. Numerous studies have correlated the levels of vesicular and protein-bound c-miRNAs with various pathologies. Moreover, secreted extracellular miRNAs can reflect molecular changes in the cells from which they originate, so their profiles differ depending on physiological and pathological conditions [63]. They can therefore be considered potential diagnostic markers for diabetes, systemic lupus erythematosus, asthma, arthritis, Alzheimer’s disease, cardiovascular diseases, various tumors, etc. [64,65,66].

In 2011, the existence of c-miRNAs in vesicular form was questioned, since only 10% of circulating microRNAs are in this form [67]; the remaining 90% circulate in association with proteins of the Argonauts family (AGO2), HDL, or other RNA-binding proteins [68]. However, it was later found that the non-vesicular forms of c-miRNA are non-specific products of physiological activity and cell death.

The basis on which one method of miRNA transport is chosen over another for delivery into the intercellular space is not clear. However, selective “packaging” of various c-miRNAs into microvesicles and exosomes has been demonstrated [68,69,70]. For example, the let-7 miRNA family in a metastatic gastric cancer line is selectively secreted into the extracellular environment exclusively by exosomes. According to the authors, this contributes to the maintenance of oncogenesis and metastasis [68]. Wang et al. showed that some human cell lines (HepG2, A549, T98, and BSEA2B) actively release miRNAs for an hour immediately after serum deprivation [71], suggesting that miRNAs are secreted in response to stress. In this experiment, the authors noticed that most of the extracellular c-miRNAs were complexed with RNA-binding proteins, not inside microvesicles or exosomes. Characteristic miRNA sequences promote interaction with the A2B1 fish nucleoprotein, while Ago2 facilitates miRNA loading into the vesicle. A mechanism for miRNA sorting based on the 3′-terminal structure was proposed [72]. These authors found that 3′-terminal-adenylated microRNAs were predominantly intracellular, while their 3′-terminal-uridylated isoforms were found in exosomes. This confirms the influence of post-transcriptional modifications on the microRNA sorting method.

miRNA is loaded into the vesicle by the interaction of characteristic miRNA motifs with the A2B1 fish nucleoprotein, which facilitates the process. Interestingly, the number of microRNAs selectively released from cells into a particular body fluid can correlate with malignant neoplasms [73]. Pigati et al. found that most of the miR-451 and miR-1246 produced by malignant mammary epithelial cells were released into the extracellular space, while most of the same miRNAs produced by normal mammary epithelial cells were retained in the cell. These results confirm a cellular selection mechanism for miRNA release and indicate differences between their extracellular and cellular profiles. This selective release makes it possible to consider c-miRNAs as biomarkers for various diseases.

The mechanisms that regulate and control exosome release into the recipient cell remain completely unknown. However, some proteins involved in this process have been identified: TAT-5 and Rab27. TAT-5 is a cell membrane-associated protein that regulates vesicle detachment [74]. Rab27 is involved in exosomal release and uptake by recipient cells [75]. Exosome release from mammalian cells is also facilitated by the ceramide pathway, which also inhibits the export of miRNAs in combination with HDL.

## 5. Analysis Methods

Most potential c-miRNA biomarkers are present in both healthy people and cancer patients, with very slight differences in expression levels. Therefore, for studying miRNA expression, initial standardization of the stage at which the material is isolated is decisive. In addition, there is still no generally accepted line of markers for all biofluids. However, an advantage of using miRNAs as biomarkers is that their expression levels are seldom affected by age, gender, body mass index (BMI), smoking status, or other characteristics underlying pathogenic potential. The most common body fluids for miRNA testing are plasma and serum, but others such as urine and saliva are also used.

When biofluids are collected to isolate miRNAs, it is important to ensure that the cellular and non-cellular fractions of the sample are properly separated. Failure to do this can cause large differences in miRNA concentration between samples because cells can contain many microRNAs. MicroRNAs specific for each biofluid have to be identified so that their levels can be used to assess contamination of samples with cellular miRNAs. Different amounts of cellular contamination can also introduce variability into the final concentrations of miRNAs detected. Therefore, highly consistent sample processing is extremely important. For example, when a sample is isolated from blood, the level of hemoglobin must be controlled to avoid sample contamination due to rupture of red blood cells. Ideally, ethylenediaminetetraacetic acid (EDTA) or citrate is used as an anticoagulant, since heparin can interfere with subsequent experimental steps such as reverse transcription and qPCR. To minimize differences between sample profiles, blood collection tubes with the same anticoagulant (no heparin) should be used. When exosomal miRNAs are examined, ultracentrifugation or a commercial kit such as ExoQuick (SBI, Palo Alto, CA, USA) is used [76]. The RNA is then extracted using commercially available methods, including phenol/guanidinium, such as TRIzol (Life Technologies, Carlsbad, CA, USA), and column-based kits, such as mirVana (Life Technologies) and miRNeasy (Qiagen, Hilden, Germany ) [77]. The same storage conditions have to be maintained for all samples as temperature and storage time can affect miRNA stability, especially during long-term storage.

Further stages in the study depend on whether the task is to examine the expression of c-miRNAs in a previously installed panel for a specific biomaterial and a number of diseases, or to discover new miRNAs.

The most common methods for studying miRNA expression are PCR with real-time reverse transcription (RT-PCR, qRT-PCR). The high specificity, sensitivity and speed of this method are advantageous, making it possible to identify a particular microRNA among hundreds of others using special microplates with specific primers. However, the short lengths of miRNAs and the lack of a common sequence for all molecules (for example, the 3′ poly(A) sequence in mRNAs) make for difficulties, though the the possibility of using conventional primers is not excluded. To overcome these difficulties, different methods with different primers and detection methods are available. One of these uses stem–loop primers to carry out reverse transcription of miRNA; the resulting cDNA is amplified with conventional primers and quantified in real time with labeled fluorophores added to the reaction medium. This method is used in platforms such as TaqMan Cards (Life Technologies). Another method involves polyadenylation of all miRNAs and adding an antisense primer with a poly(T) sequence at both the 5′ and 3′ ends, followed by reverse transcription and amplification together with the sense primer. Amplification products are detected using the SYBR Green fluorescent dye, the fluorescence intensity of which is increased about 100-fold when it is incorporated into a double-stranded structure. Thus, a particular miRNA can be quantified absolutely [24]. The main problem with this method remains the normalization of microRNA values to the appropriate endogenous control. There are many panels of endogenous miRNA genes, depending on the biomaterial under study, that maintain constant levels under various conditions and serve as controls for miRNA quantification. For example, reference genes *U6*, *RNU43*, *RNU44*, *RNU48*, and *miR-16* are used to normalize data on the number of miRNAs in blood serum. *RNU43* is used in combination with *RNU1-4* or *miR-16* with *miR-30e* as a reference in urological cancers. Xi Chen and co-author identified a new panel of *let-7d*, *let-7g*, and *let-7i* genes as a benchmark for normalizing serum miRNA, which, according to their study, is statistically superior to those previously used [78]. Lushui Wana et al. [79] identified another panel of the most stable reference genes for normalizing the results for serum miRNAs in bladder cancer (BC), which comprised the genes *hsa-miR-193a-5p* and *hsa-miR-16-5p*.

When new profiles of c-miRNA markers for various diseases are sought, samples of them are obtained from biological material and then sequenced. For this purpose, either next-generation sequencing (NGS) or microarray analysis is used. NGS combines methods for determining the nucleotide sequence of DNA or RNA, making it possible to identify several regions of the genome simultaneously. NGS is accomplished by repeated cycles of polymerase-induced chain elongation or multiple ligation of oligonucleotides. Up to hundreds of megabases and gigabases of nucleotide sequences can be generated in one working cycle. The advantage of NGS is the ability to identify new miRNAs, but the system is less cost-effective and less efficient than microarrays.

Microarray analysis allows several thousand microRNAs to be analyzed simultaneously. The microRNA samples are labeled with fluorescent probes, then further hybridized with samples of hundreds or thousands of microRNAs covalently immobilized on a solid support (microchip), followed by sample detection. To standardize the melting temperatures of the miRNAs, LNA (Locked Nucleic Acid) sequences are used, which are included in the hybridization probes. This approach helps to reduce differences in melting temperature between probes and also makes it possible to identify microRNAs with similar profiles. However, this method for quantitative analysis can cause technical difficulties, so it is often combined with RT-PCR.

One important task is to identify the expression signatures of miRNAs in the biomaterial studied. These have diagnostic value for the disease under investigation. Xumei Jiang et al. [80] examined a panel of six miRNAs (*miR-152*, *miR-148b-3p*, *miR-3187-3p*, *miR-15b-5p*, *miR-27a-3p*, and *miR-30a-5p*) to identify their expression signature in blood serum from breast cancer patients. The authors showed that the serum miRNA signature can be of significant diagnostic value, and the miR-152 expression level can elucidate the risk of recurrence of non-muscle-invasive breast cancer. Lutao Du and colleagues identified a panel of seven miRNAs in urine (*miR-7-5p*, *miR-22-3p*, *miR-29a-3p*, *miR-126-5p*, *miR-200a-3p*, *miR-375*, and *miR-423-5p*) for diagnosing and predicting bladder cancer recurrence [81]. Ghorbanmehr and colleagues identified three other miRNA markers, *miR-21-5p*, *miR-141-3p*, and *miR-205-5p*, which are expressed differently in the urine of patients with bladder and prostate cancers [82]. Research on this theme continues to be productive, and it is obvious that c-miRNAs in bioliquids have great potential as a basis for developing non-invasive test systems for diagnosing various diseases.

## 6. Advantages and Disadvantages of miRNA as a Disease Predictor

Although the class of microRNAs was discovered relatively recently [2], they have been successfully studied for more than 10 years as biomarkers for various diseases. The expression of these molecules changes in cardiovascular diseases, tuberculosis, oncology, Alzheimer’s disease, epilepsy, ischemic stroke, and many other pathologies [83]. The advantage of miRNAs over other known markers is their easy accessibility, i.e., they can be detected in any body fluid, including saliva, urine, and breast milk. Their high stability is associated with encapsulation in lipid vacuoles or complexation with proteins, which protects them from denaturation.

Another advantage of c-miRNAs is their sensitivity. They can indicate a disease before the clinical picture is manifest (during the latent period), and the profile can differ depending on the degree and severity of the disease, which is especially important for determining the stage of an oncological disease [84] and for personalized therapy. Tak Fan et al. showed the predictive power of miRNAs for the effectiveness of therapy for hepatitis virus [85].

Despite the rapid growth of knowledge about c-miRNAs, lines of these markers for individual pathologies have not yet been developed as standards; each individual miRNA has a wide and often non-specific spectrum of action. In addition, c-miRNA signatures differ depending on the biofluid in which they are detected. However, these problems can be solved if a clear algorithm for selecting markers is created, which is possible when the database of detected miRs, which is rapidly being replenished with new samples, has been sufficiently expanded. Furthermore, no standard protocol for c-miRNA detection has been approved so far. A protocol should include methods for isolation and storage and the detection technology itself. However, all these obstacles to using c-miRNAs as biomarkers for various diseases can be overcome by further studies.

## 7. The Use of miRNAs as Targets in the Treatment of Diseases

The therapeutic approach to using miRNAs can be divided into two categories: (1) miRNA inhibition therapy, when they are overexpressed [86]; and (2) miRNA replacement therapy, when they are repressed. The former approach uses an anti-miRNA or miRNA inhibitor consisting of a single-stranded oligonucleotide with a sequence complementary to the mature miRNA. The latter approach uses synthetic miRNA mimics with a sequence identical to that of endogenous mature miRNA. The principle is that such miRNAs are introduced into cells as an exogenous supplementary source. They are delivered in either plasmids or viruses for further expression, or by modifying the miRNA molecules themselves to stabilize them in the cell’s internal environment. The approach to treating diseases at the translational level using miRNAs has advantages, since it makes it possible to “target” a specific gene and thereby inhibit it highly specifically without affecting the genetic material of the host cell [87]. Already, drugs based on miRNAs are undergoing clinical trials.

## 8. Targeted miRNA Delivery Strategies In Vivo 

To control the silencing of target genes, miRNA molecules need to leave the endosome and immediately enter the cytoplasm, where they bind to the RISC complex and cleave the complementary mRNA pointwise.

**Figure 3:**
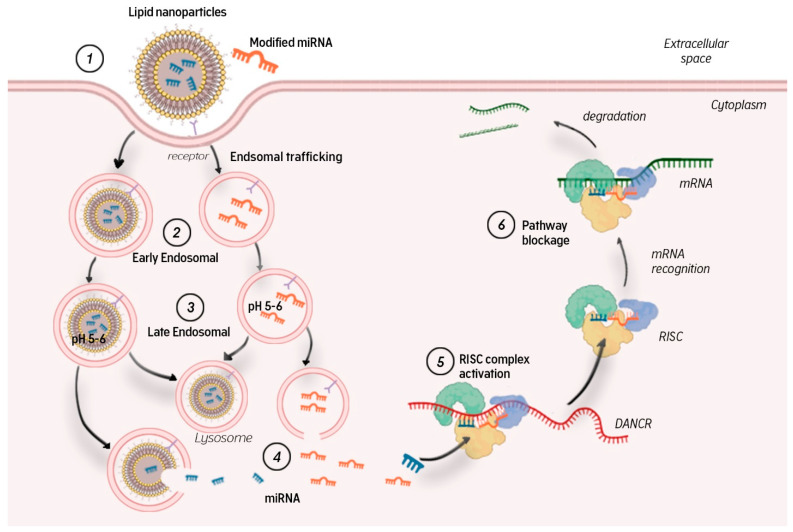
Transport of microRNA inside the cell (description in the article).

MicroRNAs in a complex of nanoparticles or modified molecules are captured by target cells through receptor-mediated endocytosis (Figure 3). Endocytic vesicles fuse and form early endosomes, which transfer their contents to late endosomes. Late endosomal vesicles have an internal pH of 5–6 owing to membrane-bound proton pump ATPases. Their contents are then transported to lysosomes, which have an even lower pH of ~4.5. Lysosomes also contain nucleases that promote miRNA degradation. To avoid this, miRNAs (free or complexed with a carrier) must exit the endosome into the cytosol, where they can bind to the RISC complex and participate in RNA interference. Endosomal yield is another obstacle to efficient miRNA delivery [13]. If this stage is overcome, the microRNA guide strand in the RISC complex interacts with complementary mRNA regions, leading to mRNA degradation and/or blocked translation.

Thus, a primary and major barrier to miRNA delivery is the plasma membrane. Being hydrophilic and negatively charged, miRNAs cannot penetrate into the cell easily. Another difficulty in using exogenous miRNAs is their short half-life in the blood owing to the nucleases therein. After the first barrier is overcome, the miRNA must be delivered to the cytoplasm bypassing the lysosome so it can bind to RISC and perform its silencing function. Therefore, the main challenge in using miRNAs as potential therapeutic agents is the development of high-precision platforms that can overcome all the difficulties of delivery and cellular uptake. The aims of these developments are: (1) to increase the residence time of miRNAs in the circulation by reducing the rate of renal clearance; (2) to protect them from serum nucleases; (3) to ensure efficient biodistribution; (4) to facilitate accurate delivery to the cytoplasm and capture by the target cell RISC system [88].

**Figure 4:**
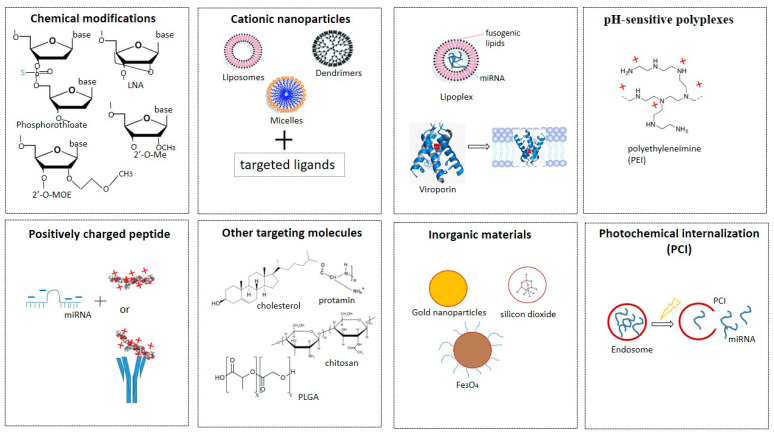
The various approaches to maintaining the stability of miRNAs in vivo and ensuring targeted delivery to cells.

To date, there have been many approaches to maintaining the stability of miRNAs in vivo and ensuring targeted delivery to cells (Figure 4). The main ones include using: phosphorothioate-containing oligonucleotides [89], 2′-O-methyl-(2′-O-Me), or 2′-O-methoxyethyl oligonucleotides (2′-O-MOE) [90], locked NA (LNA), oligonucleotides [90], peptide NA (PNA) [91], fluorine derivatives (FANA and 2′-F), and others [21,92]) for chemical modifications of miRNAs.

1. Phosphorothioate-containing oligonucleotides [89], 2′-O-methyl-(2′-O-Me), or 2′-O-methoxyethyl oligonucleotides (2′-O-MOE) [90], locked NA (LNA), oligonucleotides [90], peptide NA (PNA) [91], fluorine derivatives (FANA and 2′-F), and others [21,92]) for chemical modifications of miRNAs.

2. *Cationic nanoparticles*, such as *liposomes, micelles, vesicles, or dendrimers*, which condense with negatively charged polynucleic acids through electrostatic interactions, increasing permeability and accumulation [93,94]. Since RNAs in liposomes enter the cell by endocytosis, they are usually used in combination with targeted ligands. The lipid nanoparticle–mRNA vaccines are now in clinical use against coronavirus disease 2019 (COVID-19) [95]. Of the many COVID-19 vaccines under development, the two vaccines are composed of mRNA strands encapsulated in lipid nanoparticles. The efficacy of these mRNA vaccines developed by BioNTech/Pfizer and Moderna is about 95%. A drawback of the current mRNA–lipid nanoparticle COVID-19 vaccines is that they have to be stored at low temperatures. Thus, developing new delivery systems for mRNA and miRNA is an important objective in modern pharmacology [96].

3. *Fusogenic lipids* to improve the delivery of NA into cells. One example is the development of a multifunctional shell-like structure for efficient delivery [97]. This structure consists of NA molecules enclosed within a liposome enriched with fusogenic lipids. Such structures are called lipoplexes. They can join and merge with anionic membranes, leading to the release of their contents into the cytoplasm. Another approach to gene delivery is to use highly hydrophobic proteins, *viroporins*, which can form channels and thereby destabilize biological membranes [98]. However, it is difficult to maintain the stability of lipoplexes. One strategy involves modifying them to protect positively charged fusogenic liposomes from interaction with serum proteins and macrophages. Kumar et al., for example, developed pH-sensitive cationic lipoplexes to replace commercially available transfection reagents of the Lipofectamine series [99]. Lipoplexes with altered ratios of molar masses of cationic pH-sensitive liposomes to pDNA (plasmid) have been developed. Lipoplexes containing pDNA have approximately 1.3-fold higher tumor transfection rates than Lipofectamine lipoplexes, indicating a superior ability to deliver genes in vivo. Studies of pDNA lipoplexes have also shown high cell viability and transfection efficiencies 5.00 times higher than those of Lipofectamine lipoplexes. Another example is the work of Guo and colleagues, who also developed biocompatible liposomes sensitive to small changes in pH [100]. They synthesized a hydrophilic, pH-sensitive polymer, polyethylene glycol orthoesther-distearoylglycerol, and used it to modify the outer surfaces of DOPE (dioleoyl-phosphatidylethanolamine) vesicles. The polymer–liposome conjugate was highly stable in serum, but most of the polymer molecules hydrolyzed rapidly as the pH was lowered, promoting aggregation and fusion of PE-rich lipid vesicles.

4. *pH-sensitive polyplexes* are used as non-viral vectors for targeted delivery of NA. These consist of positively charged polymers (polycations) and negatively charged NA molecules, which they condense into complexes, ensuring their stability and protection from nucleases [101]. The best-known example is polyethyleneimine (PEI) [102]. In a physiological environment, PEI is positively charged owing to the protonation of amino groups so it can be used for NA condensation. The polyplexes formed by PEI and NA usually retain a net positive charge, facilitating interaction with negatively charged polysaccharides on the cell surface. It is assumed that after interaction with the cell surface, the complexes undergo endocytosis. Examples of this group of miRNA delivery agents used successfully are PEI [103], PEI short-arm polyurethane [104], and dendrimers [105,106,107].

5. *Positively charged* *peptides* (e.g., polyarginine) or *proteins associated with an antibody or ligand* can be combined with negatively charged miRNAs. The same approach (attachment of short peptides) is used as an alternative strategy for modifying lipoplexes or polyplexes to improve endosomal miRNA release [108].

6. Targeting molecules such as *aptamers, ligands* (for example, a cholesterol fragment) or *natural polymers* (chitosan, protamine, atellocatagen) [109,110,111] and *PLGA polyconjugates* (poly(lactic-co-glycolic acid)) for conjugation with miRNAs. The latter are water-insoluble polymers that protect miRNAs from degradation, have a high loading capacity, and allow for many surface modifications to achieve the best pharmacodynamic characteristics [112,113,114].

7. *Inorganic materials* (gold nanoparticles, silicon dioxide, magnetic particles) [115].

8. *Photochemical internalization technology* is used to optimize the release of endocytosed macromolecules into the cytosol. Endocytosed photosensitive molecules (photosensitizers) are activated by light to release the contents of endocytic vesicles before they enter lysosomes [116]. Photosensitizers are natural or synthetic compounds that when exposed to light stimulate the formation of reactive oxygen species, primarily singlet oxygen, which, having a short lifespan and a limited range of destruction, damage only the endosomal membrane, promoting the release of contents without affecting other organelles.

## 9. Conclusions

As can be seen, there is already a huge range of approaches to solve the problems of miRNA delivery to a strictly targeted target. Nevertheless, research in this direction continues.

The discovery of miRNAs was a scientific breakthrough, and the study of their functional potential opened the possibility of influencing protein synthesis at the gene level. Moreover, miRNAs in this class have proved to be highly sensitive biomarkers for various diseases, making it possible not only to detect a disease at early asymptomatic stages, but also to predict therapeutic efficacy. In the future, this will help in the development of personalized medicine.

## Data Availability

The data presented in this study are available on request from the corresponding author without any restrictions.

## Abbreviations

c-miRNA	Circulating microRNA
siRNA	Small interfering RNA
mnRNA	Small non-coding RNA
cDNA	Complementary DNA
PCR	Polymerase chain reaction
